# Evagination of Cells Controls Bio-Silica Formation and Maturation during Spicule Formation in Sponges

**DOI:** 10.1371/journal.pone.0020523

**Published:** 2011-06-02

**Authors:** Xiaohong Wang, Matthias Wiens, Heinz C. Schröder, Ute Schloßmacher, Dario Pisignano, Klaus Peter Jochum, Werner E. G. Müller

**Affiliations:** 1 National Research Center for Geoanalysis, Beijing, China; 2 European Research Council Advanced Grant Research Group, Institute for Physiological Chemistry, University Medical Center of the Johannes Gutenberg University Mainz, Mainz, Germany; 3 Dipartimento di Ingegneria dell'Innovazione, Università del Salento and National Nanotechnology Laboratory of CNR-Istituto Nanoscienze, Lecce, Italy; 4 Max Planck Institute for Chemistry, Mainz, Germany; The University of Manchester, United Kingdom

## Abstract

The enzymatic-silicatein mediated formation of the skeletal elements, the spicules of siliceous sponges starts intracellularly and is completed extracellularly. With *Suberites domuncula* we show that the axial growth of the spicules proceeds in three phases: (I) formation of an axial canal; (II) evagination of a cell process into the axial canal, and (III) assembly of the axial filament composed of silicatein. During these phases the core part of the spicule is synthesized. Silicatein and its substrate silicate are stored in silicasomes, found both inside and outside of the cellular extension within the axial canal, as well as all around the spicule. The membranes of the silicasomes are interspersed by pores of ≈2 nm that are likely associated with aquaporin channels which are implicated in the hardening of the initial bio-silica products formed by silicatein. We can summarize the sequence of events that govern spicule formation as follows: differential genetic readout (of silicatein) → fractal association of the silicateins → evagination of cells by hydro-mechanical forces into the axial canal → and finally processive bio-silica polycondensation around the axial canal. We termed this process, occurring sequentially or in parallel, bio-inorganic self-organization.

## Introduction

The siliceous skeletal elements of the sponges [phylum: Porifera], termed spicules, possess several unique features which distinguish them from the skeletal elements found in other Metazoa. They are made of silica [(SiO_2_)*_n_*] instead of Ca-based minerals [1] with an unparalleled precision, giving rise to species-specific complex structures [2]. These genetically controlled and biologically produced structures are formed at ambient, mild physiological conditions, without high temperatures, pressures, or caustic chemicals [3]. The spicules are the critical structural determinant that controls the morphology of the sponges [4], [5]. In the center of the spicules lies a 0.5–4.0 µm wide axial canal which harbors the organic axial filament [6], [7]. Since its discovery the axial filament has been considered to be a template that controls the morphology of the spicules [8]. A major step towards an understanding of the genetically controlled morphogenesis of sponges was the identification of the structural protein of the spicules, termed silicatein which is located in the axial filament [9] as well as on the surface of the spicules [10]. Silicatein is an enzyme which forms the bio-silica required for the construction of the sponge spicules [11]–[14]. The formation of spicules is a rapid process, which lasts for a spicule with a length of 190 µm and a diameter of 6 to 8 µm at 21°C only 40 hrs [15]. Because of this high growth rate it remained unclear for a long time if spicule formation starts intra- or extracellularly [16], [17].

Detailed cell biological and biochemical studies on the intracellular spicule formation have been performed with the sponge *Suberites domuncula* [18], [19]. These studies became possible since the establishment of a suitable cell culture system (the primmorphs) from *S. domuncula*, which allowed time-lapse developmental studies of spicule formation under controlled conditions [20]. The 3D-cell culture is composed of proliferating and differentiating stem cells, and of sclerocytes that initially form the spicules [21]. In these studies we described that silicatein-mediated spicule growth proceeds in two directions. Firstly, in axial, longitudinal direction in which the growth of the spicule is driven by the 23 kDa processed form of silicatein. Secondly, the radial thickening of the spicules, their appositional growth, occurs after extrusion of the spicules into the extracellular space. Accumulation of silica on the surface of the growing spicule in centripetal direction is mediated by the 34.7 kDa silicatein [10], [18]. This form of silicatein is distinguished from the 23 kDa enzyme by the presence of the N-terminal pro-peptide sequence that is presumably cleaved off autocatalytically immediately before the onset of bio-silica synthesis [18]. In this study no conclusive evidence has been obtained for the existence of collagen either in the axial filament or on the surface of the spicules that would be causatively involved in bio-silica formation, as has been speculated [22].

Earlier studies on silicatein-driven spicule synthesis did not answer the question of how elongation of the spicule in axial direction occurs [18], [19]. Two observations have been published which showed that even after the release of the spicules into the extracellular space the axial filament undergoes maturation steps. These data revealed that thereby an alteration from a less compact organization of the organic components within the axial canal, which also includes membraneous structures, to a compact axial filament occurs [18], [19]. In support it was found that during maturation of the spicules the diameter of the axial canal decreases from approximately 4 µm to 0.5 µm. The release of the intracellularly formed spicules, their extrusion into the extracellular space, was assumed to be facilitated by spicule associated filaments [18], [23]. The final shaping of the spicules surely occurs extracellularly while growing from 6 µm to 150–320 µm. It should be stressed here that in sponges, in contrast to the more evolved metazoan taxa, the cells within the body are only loosely attached to each other by cell-cell contacts, and are positioned within the tissue by stronger cell-matrix interactions [reviewed in: 24]. In the extracellular space of the sponge tissue galectin is the dominant protein, and functions there as a structural protein [25]. Galectin is a molecule that can, in the presence of Ca^2+^, turn from the sol into the gel state, and then associate with silicatein molecules together; these two components form an organic cylinder around the surface of a growing spicule into which bio-silica is finally deposited [10]. A collagen cast is arranged around that organic cylinder comprising the moldable bio-silica and models the growing spicule [26].

In the present study we give for the first time experimental evidence for the existence of one cellular process originating from a spicule-forming cell, a sclerocyte, into the axial canal of a spicule. Such an extension evaginates into the axial canal where it controls axial growth and releases silicasomes [27]. From these vesicles silicatein and silicic acid are transported into the extracellular space resulting in the deposition of bio-silica at the inner surface of the siliceous mantel. During maturation of the spicule in the extracellular space two polycondensation/deposition processes occur that are spatially separated; first on the outer surface of the growing spicule and second on the inner surface of the spicule [the wall of the axial canal]. Thus, two polycondensation reactions run in parallel, first, centripetally directed polycondensation, resulting in the formation of the bio-silica core around the axial filament within the axial canal, and second, centrifugally directed polycondensation on the outer surface of the spicule, leading to the synthesis of the bio-silica shell of the spicule. During these processes the cell extension elongates by an energy-consuming process which is very likely fed by ATP cleavage through an arginine kinase. In *S. domuncula* we found that the gene encoding an arginine kinase is induced by silicate [28]. In addition to silicatein and arginine kinase, whose presence in the axial canal has been demonstrated by immunofluorescence staining, a third protein, aquaporin-8 (to be published; EMBL accession number FR773712) that had been implicated in the maturation of bio-silica, was recently identified by specific antibodies. Aquaporins are channels interspersed in the cell membrane, which regulate the flow of water in general [29], and very likely translocate the reaction water that is released during the polycondensation reaction; thereby they facilitate the maturation/ageing process of the bio-silica material [30]).

Based on the presented data we propose a model to describe the experimental findings of bio-silica formation in the core of the spicules. Our data strongly support the view that axial spicule growth is driven by the elongation of cell processes into the axial canal, and is mediated by silicatein and silica that are released from the silicasomes.

## Materials and methods

### Sponge, primmorphs and spicules

Specimens of *S. domuncula* (Porifera, Demospongiae, Hadromerida) were collected in the Northern Adriatic near Rovinj (Croatia), and then kept in aquaria in Mainz (Germany) at 17°C for more than 5 months. *S. domuncula* synthesizes monaxonal spicules, primarily tylostyles, measuring 150–320 µm in length and 6.14 to 6.57 µm in diameter. While one end of the spicules is pointed, the other is blunt with a globular swollen knob [31]. The rarer oxeas, with two pointed ends, can reach sizes of up to 430 µm.

Primmorphs were prepared from *S. domuncula* as described [20]. These 3D cell aggregates were cultivated for 10 days in natural seawater (Sigma, Taufkirchen; Germany) supplemented with 1% RPMI 1640 medium (Sigma). Spicules were isolated by soaking 10 days old primmorphs in nitric acid/sulfuric acid (1 ∶ 4 v/v) for 2 days, followed by washing in distilled water until the pH value was 6.

### Electron microscopy - immunocytochemical procedure

For transmission electron microscopy [TEM] analysis primmorph sections were transferred onto coated copper grids and analyzed with a Tecnai 12 microscope (FEI Electron Optics, Eindhoven; Netherlands). Slices were prepared as described [18] by dehydration with ethanol, followed by fixation in propylene oxide/araldite and embedding in araldite. Cutting to 60-nm ultrathin slices was performed with an Ultracut S ultramicrotome (Leica, Wetzlar; Germany). Electron immunogold labeling/TEM analysis was performed with slices treated in glutaraldehyde/paraformaldehyde buffered in phosphate buffer [PBS] [18]. The samples were reacted with one of the following primary polyclonal antibodies (PoAb), with anti-silicatein-α (PAb-aSILIC_SUBDO; 1∶1,000; [18]) for 12 hrs at 4°C, with anti-aquaporin-8, raised against the recombinant protein from the *S. domuncula* cDNA (PoAb-aAQP_SUBDO: 1∶1,000; accession number FR773712), or with anti-arginine kinase PoAb, raised against the *S. domuncula* recombinant protein (PoAb-aAK_SUBDO; 1∶1,000; accession number AJ744770) [28]. Subsequently, the sections were incubated with a 1∶100 dilution of the secondary antibody (1.4-nm nanogold anti-rabbit IgG; diluted 1∶200) obtained from Nanoprobes (Yapbank, NY). After rinsing with PBS the samples were exposed to silver to enhance the immunocomplexes. In controls, preimmune serum was used instead of the primary antibodies. High-resolution scanning electron microscopy (SEM) analyses of the samples were performed with a Gemini Leo 1530 high-resolution field emission scanning electron microscope (Zeiss, Oberkochen; Germany).

### Immunohistology

The detailed procedure was given previously [18]. In brief, tissue samples were embedded and 8-µm-thick frozen sections were cut. The cryosections were fixed and then reacted with the first antibody (1∶1,000 dilution). After blocking with nonfat dry milk/bovine serum albumin the samples were subjected to the second antibody (Cy5-conjugated F(ab′)2 goat anti-rabbit IgG; Jackson ImmunoResearch, Cambridgshire, UK; at a 1∶2,000 dilution). Immunofluorescence images were taken with an Olympus AHBT3 light microscope, together with an AH3-RFC reflected light fluorescence attachment at an emission wave-length of 670 nm (filter G). The slices had been counterstained with 4′-6-diamidino-2-phenylindole (DAPI; Sigma) to visualize the cell nuclei and inspected at 420 nm.

### Analysis energy dispersive X-ray spectroscopy (EDX)

Primmorph samples were embedded in epoxy resin as described [32]. Then the aggregates/primmorphs were cut to about 200 nm thick slices with an Ultra-Microtome (Leica EM; Leica Microsystems Japan, Tokyo; Japan). Those sections were placed onto a grid and analyzed (see above). EDX analyses were performed with a Philips 420 TEM and a Nova 600 NanoLab SEM/FIB, equipped with an EDAX Division EDX analyzer.

## Results

### Growth of the spicules

**Figure 1:**
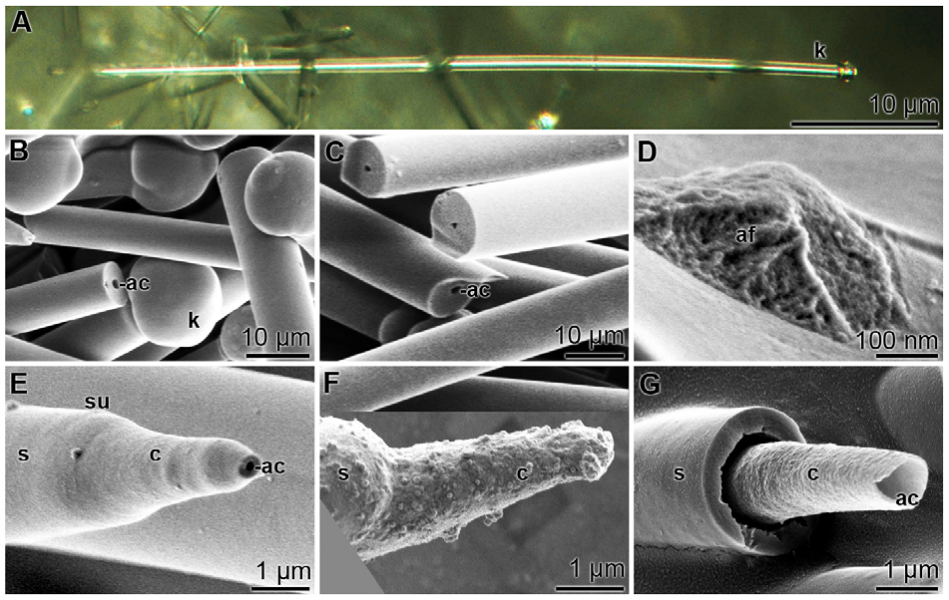
Different microscopic techniques reveal the morphology of *S. domuncula* spicules; (A) light microscopy, (B–G) SEM. (**A**) A tylostyle, a monaxonal rod with a terminal knob (k) on one side and a pointed tip at the other end. **B** and **C** show the blunt end with the knob (k) and some broken spicules exhibiting the axial canal (ac). (**D**) A broken spicule displaying the bulgy material constituting the axial filament (af). (**E**) A developing spicule with a progressively growing tip; the central core shell (c) around the axial canal (ac), and the final silica shell (s). The surface (su) of the spicule is marked. (**F**) Mineral deposits on a growing spicule causing the granular surface of its core (c) and shell (s) regions. (**G**) A broken spicule, displaying the internal mineral core (c) surrounding the axial canal (ac), and the outer shell (s).

The prevalent spicule type found both in tissue and in primmorphs from *S. domuncula* were tylostyles (Fig. 1A); the monaxonal rods displayed one blunt end, appearing as a knob, and one pointed tip. The length of the shaft was 150–320 µm. The globular knobs emerging from the shaft had sizes between 6.5 and 9.2 µm (Fig. 1B). Each monaxonal rod comprised a central axial canal (Fig. 1B and C). In the axial canal an organic axial filament existed that displayed a granulated structure (Fig. 1D). The bulgy material constituting the axial filament tightly filled the axial canal.

As outlined in the “Introduction”, the synthesis of the spicules is a rapid process and in turn developing spicules can only be seen very scarcely in tissue from adult animals. However, during cultivation of primmorphs starting from single cell suspension growing spicules can be seen more frequently. In Fig. 1 E and Fig. 1G such spicules are shown. The progressively forming spicules showed a distinct zonation, into a protruding solid central core with a diameter of 2.5–3.2 µm and a surrounding thicker outer mantel, the shell of the spicule (Fig. 1E to Fig. 1G). In about 15% of those spicules the surfaces were not smooth but grainy suggesting fresh deposition of minerals (Fig. 1F). Cross fractures through growing spicules showed that the internal mineral core, which surrounded the axial canal was detached from the outer shell (Fig. 1G).

**Figure 2:**
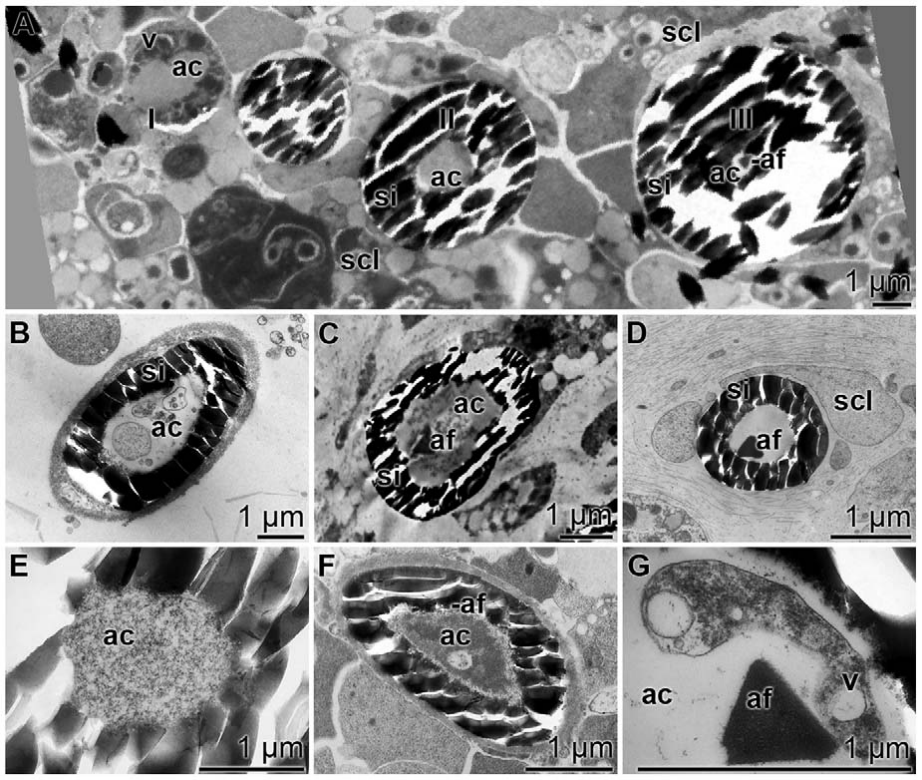
TEM images of the axial canal of spicules in primmorphs at different developmental stages. (**A**) The three major developmental phases during spicule formation: phase I: primordial spicule comprising a large axial canal (ac) which is surrounded by an organic cylinder enclosing vesicles (v). phase II: the spicule shows the siliceous mantel (si) surrounding the small axial canal (ac) devoid of a pronounced axial filament. phase III: such spicules have a small sized axial canal and a distinct axial filament (af); scl, sclerocyte. (**B**) Spicule in phase II. Membraneous structures can be resolved in the axial canal (ac) which is surrounded by the silica mantel (si). (**C**) Spicule between phases II and III showing in the axial canal a well developed axial filament (af) embedded in membranous structures, which is surrounded by the silica mantel (si) (**D**) Mature spicule with an axial filament (af) without any cellular structures. One surrounding sclerocyte is marked (scl). (**E**) Axial canal (ac), close to the apex of the spicule, comprising a homogenous granular material. (**F**) Spicule with an axial canal at phase II/III. The axial canal (ac) comprises a growing axial filament (af). (**G**) Intermediate spicule phase between II and III comprising an axial canal (ac) showing cellular structures with vesicles (v) and one axial filament (af).

Cross sections through growing spicules revealed a change in the widths of the axial canals and also of the structures in the axial filament. Fig. 2A shows the three growth phases of the spicules in a TEM image: they are labeled in this figure with I to III. Phase I: In the initial stage the diameter of the central axial canal was ≈2.5 µm and it comprised an organic material. The axial canal was surrounded by an organic cylinder that included several vesicles, filled with electron dense material (Fig. 2A phase I). The average size of these vesicles that have previously been identified as silicasomes was 150 nm [27]. These initial spicules were not yet surrounded by a silica mantel. Phase II: During phase II spicules started to form a siliceous mantel. The diameters of the spicules in this phase were between 2–4 µm and the axial filament had not yet been developed (Fig. 2A phase II and Fig. 2B). However, in this phase cell processes became visible (Fig. 2B). In Phase III an axial filament was seen which was composed of an electron-dense material (Fig. 2A phase III and Fig. 2C). The axial filament was embedded in an organic matrix that comprised in the early stages membraneous fragments, originating from the cell processes (Fig. 2C), that were absent in a later stage. In the final stage (phase III) only the axial filament could be identified in the axial canal (Fig. 2D). Subsequently, the diameter of the axial canal shrunk to a size of 2 to 0.5 µm. During maturation the axial filament changed its form from spindle-like (Fig. 2F) to triangular (Fig. 2A [III]; Fig. 2G). Besides the three main phases I to III also intermediary stages have been found. Examples are Fig. 2C, Fig. 2F and Fig. 2G being in phase II/III. In some cross sections no axial filament and no cellular structures were seen (Fig. 2E). Those cross sections had been performed close to the apex of the axial canal (see below).

### Evagination of cells

**Figure 3:**
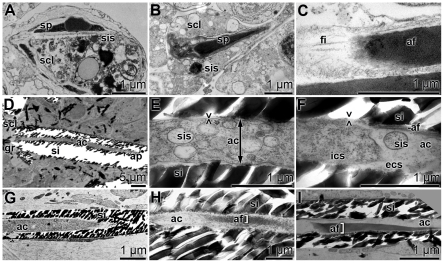
Longitudinal sections through spicules, showing the process of evagination of cells into the axial canals (ac) of the spicules, as elucidated by TEM. (**A** and **B**) Intracellular onset of spicule (sp) formation in sclerocytes (scl). The growing spicules are surrounded by silicasomes (sis). (**C**) A developing axial filament (af), that possesses at its blunt end several filaments (fi). (**D**) A longitudinal section through a spicule with its axial canal (ac). The axial canal is surrounded by a silica mantel (si). The silica fragments, that occurred during cutting of the primmorphs, were partially removed. The axial canal is closed at the apex (ap) of the spicule, while it is open at the end that is associated with the sclerocyte (scl). There the growth zone of the spicule (gr) exists. (**E** and **F**) The middle part of that spicule shows in the axial canal (ac) many silicasomes (sis). Those vesicles are surrounded by a membrane, which we perceive as cell membrane (><). Furthermore, the intracellular space (ics) and the developing axial filament (af) are marked. In the extracellular space (ecs) within the axial canal (ac) a silicasome (sis) can be identified. These images were taken close to the apex of the axial canal (ac). (**G**) Axial canal at the apex (ac), comprising no membranous structures and no axial filament. (**H** and **I**) An axial filament (af) in the extracellular space within the axial canal (ac).

The spicule formation started intracellularly in vesicles within sclerocytes ([18]; Fig. 3A and Fig. 3B). There, 1.5 to 2.0 µm long spicules were surrounded by a membrane. Adjacent 100–300 nm large silicasomes were found that were filled with electron-dense material. Near the blunt ends of the spicules the intracellularly formed axial filaments were associated with other thin filaments (diameters of approximately 40–50 nm) [28] of yet undetermined nature (Fig. 3C). From longitudinal sections through spicules strong evidence could be obtained that cellular structures protrude into the spicules, specifically into their axial canal (Fig. 3D to Fig. 3F). The longitudinal section through a complete monaxonal 50 µm long spicule showed that the axial canal was closed at one end with a siliceous layer, while the other end of the channel was open and associated with a sclerocyte (Fig. 3D). From the TEM images taken we have strong reasons to accept that the open end of the axial canal is connected with the sclerocyte via one cell protrusion. Consequently this site would represent the growth region of the spicule. Two images have been taken at higher magnification (*i*) from the middle part (Fig. 3E) and (*ii*) from the region close to the apex (Fig. 3F). (i) In the middle of the growing spicule densely packed vesicles were identified, which were surrounded by a membrane that likely represents the cell membrane (Fig. 3E). The vesicles that existed in large number were considered to be silicasomes. (*ii*) At the more terminal end, closer to the apex of the axial canal, the cell membrane ended before the silica rim leaving an open extracellular space. There, “extracellular” vacuoles, silicasomes, of sizes between 50 and 200 nm existed (Fig. 3F). In this image, the end of the cell process is seen. In the extracellular space between the axial canal and the cell extension a developing axial filament is seen. An overview of the apex region of the axial canal is shown in Fig. 3G. Figs. 3H and Fig. 3I show further examples of spicules with growing axial filaments in the extracellular space of the axial canal.

From these data we propose that into each growing spicule one cell process protrudes. At the entrance of the cellular extension into the axial canal the diameter of the canal was much larger (1 to 3 µm) while it decreased steadily in size towards the apex which was surrounded by the siliceous mantel.

### EDX analyses

**Figure 4:**
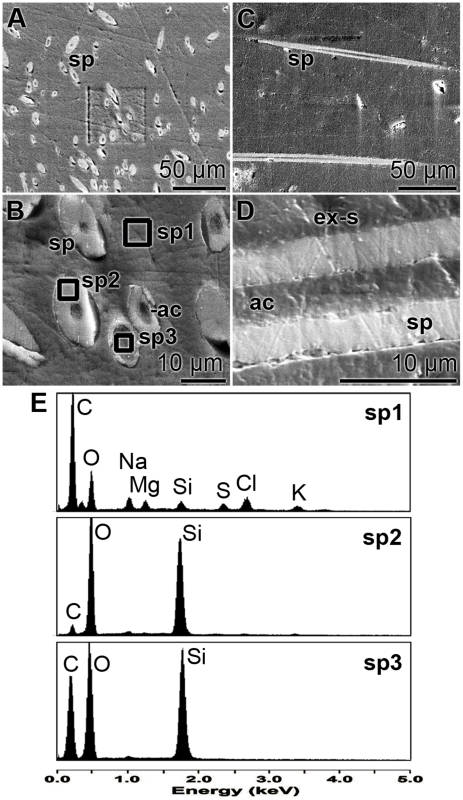
EDX analysess were performed of cross sections through primmorphs displaying growing spicules (sp). The sections were made in transversal (**A** and **B**) and in longitudinal orientation (**C** and **D**); SEM analyses. (**E**) EDX spectra from areas in the extra-spicular space (ex-s) [**sp1**], within the siliceous mantel of the spicule [**sp2**], and the region across the axial canal (ac) of one spicule [**sp3**]. The areas where the spectroscopic analysis were performed are marked in (B).

EDX analyses were performed to prove whether the organic material existing in the axial canal contains silicic acid which could serve as substrate for the polycondensation reaction catalyzed by silicatein. Therefore, primmorphs were sectioned and especially areas where spicules were cut transversely or longitudinally were selected for the spectroscopic analyses. Figs. 4A and Fig. 4B show such areas with cross-sectioned spicules.

Areas from the extra-spicular space (spectrum 1), from the siliceous mantel surrounding an axial canal (spectrum 2), and finally from the axial canal itself (spectrum 3) were selected and analyzed by EDX. The spectra show (Fig. 4E) that in the extra-spicular space the silicon [Si] signal was low (upper panel), while in the siliceous mantel region almost exclusively Si and oxygen [O] could be detected (middle panel). In the axial canal carbon appeared as a major peak, besides Si and O (lower panel). In a parallel series of experiments, longitudinal sections were selected (Fig. 4C and Fig. 4D). Also there the EDX spectroscopic data showed that the axial canal comprised C, besides si and O (not shown).

### Ultrastructure of the vesicles present in the axial canal

**Figure 5:**
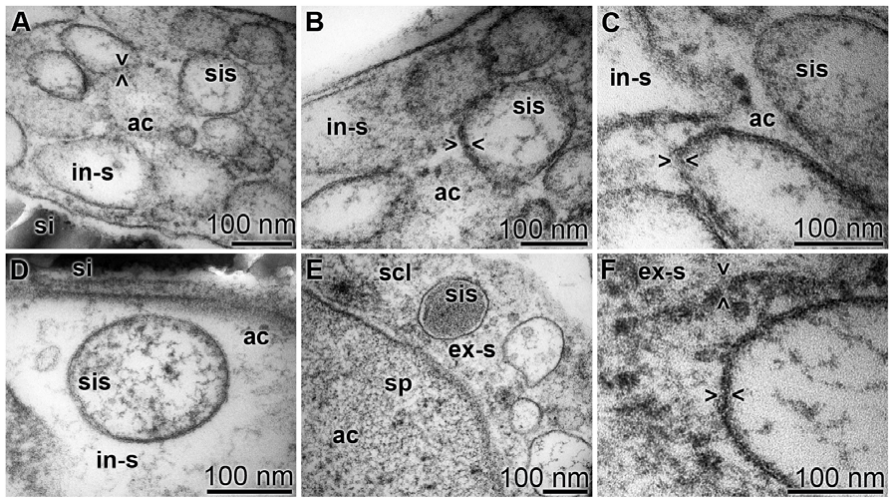
The ultrastructure of the vesicles/silicasomes was analyzed by TEM. Intra-spicular (in-s) and extra-spicular (ex-s) regions, comprising silicasomes (sis), were studied. (**A** to **D**) The intra-spicular silicasomes were found (A to C) densely packed within the cell extensions protruding into the axial canal (ac) and also (D) outside of the cell extensions. (**D**) One silicasome (sis), identified in the extracellular space within the axial canal (ac), was surrounded by the silica mantel (si). (**E** and **F**) Silicasomes found on the surface of the spicules, in the extra-spicular space (ex-s). (E) Some of the silicasomes had an electron-dense content; scl, sclerocyte. (F) In all silicasomes (sis) the membrane was perforated; some of the pores are marked (><).

The ultrastructural analysis by TEM revealed that vesicles, silicasomes, were abundant within the axial canal of developing spicules. Those vesicles displayed pores in their membranes (Fig. 5). The 50–200 nm large vesicles were densely packed in the cellular processes (Fig. 5A). Silicasomes can be filled either with electron-dense or electron-poor material. It seemed that silicasomes that occurred closer to the silica mantel were electron-dense (Fig. 5E). At high magnification, on the <100 nm scale, it could be seen that the membranes of the vesicles were not homogeneous but interrupted by pores (Fig. 5B). The membrane pores were 10 to 15 nm apart. This distance between the pores was largely constant both for the silicasomes existing in the intra-spicular (Fig. 5B to Fig. 5D) and those in the extra-spicular space (Fig. 5E and Fig. 5F). The dimension of the pores can only be approximated with 1–2 nm (Fig. 5B and Fig. 5F).

### Light immunocytochemical studies

**Figure 6:**
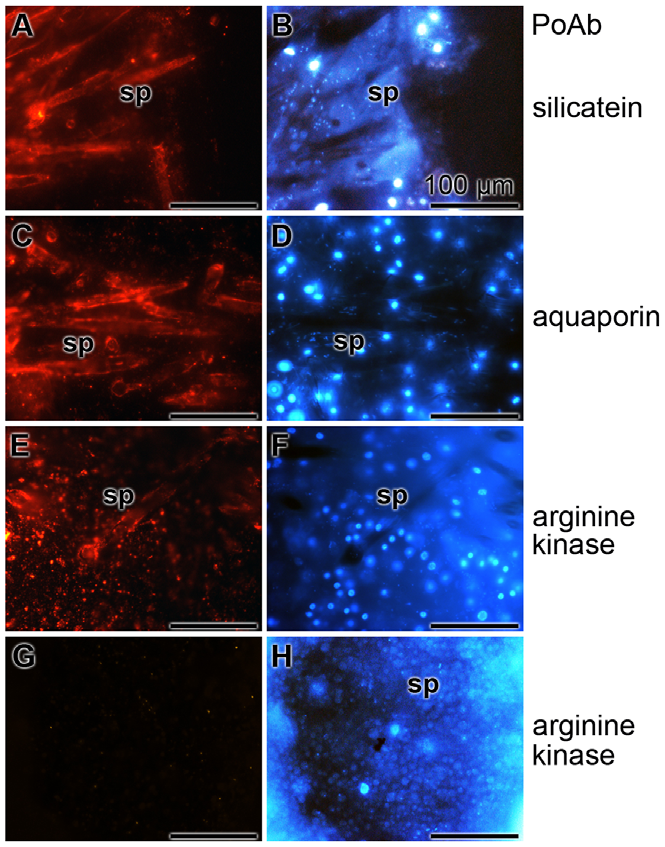
Immunostaining of cryosections through primmorphs of *S. domuncula* showed growing spicules (sp). The 8-µm thick frozen sections were reacted with one of the following polyclonal antibodies; with anti-silicatein PAb-aSILIC_SUBDO (**A** and **B**), with anti-aquaporin (PoAb-aAQP_SUBDO) (**C** and **D**), or with anti-arginine kinase (PoAb-aAK_SUBDO). (**E** and **F**) In one control series, the slices were reacted with preimmune serum from an animal used for immunization with aquaporin (**G** and **H**). The immunocomplexes were visualized with red florescent light, while the corresponding DAPI patterns were recorded with blue fluorescence light. All size bars represent 100 µm.

Cryosections were prepared and reacted with antibodies raised against silicatein, aquaporin and arginine kinase. The reaction of the anti-silicatein antibody was very strong on the surfaces and also in the center of the spicules (Fig. 6A). Since the spicules are closed, not all antibodies could reach the axial filament residing in the axial canal. Likewise intense was the reaction of anti-aquaporin with antigens on the slices (Fig. 6C). Again the surface and the axial canal were stained. Finally also anti-arginine kinase gave a strong reaction with the structures, but the staining pattern was more diffuse (Fig. 6E). The staining of these antibodies was specific, since all three preimmune sera failed to stain any structure. The images for the reaction with preimmune serum collected prior to the immunization with arginine kinase are shown here (Fig. 6G). The slices had been counterstained with DAPI allowing a staining of the nuclei (Fig. 6B, D, F and H).

### TEM-immunocytochemical procedure

**Figure 7:**
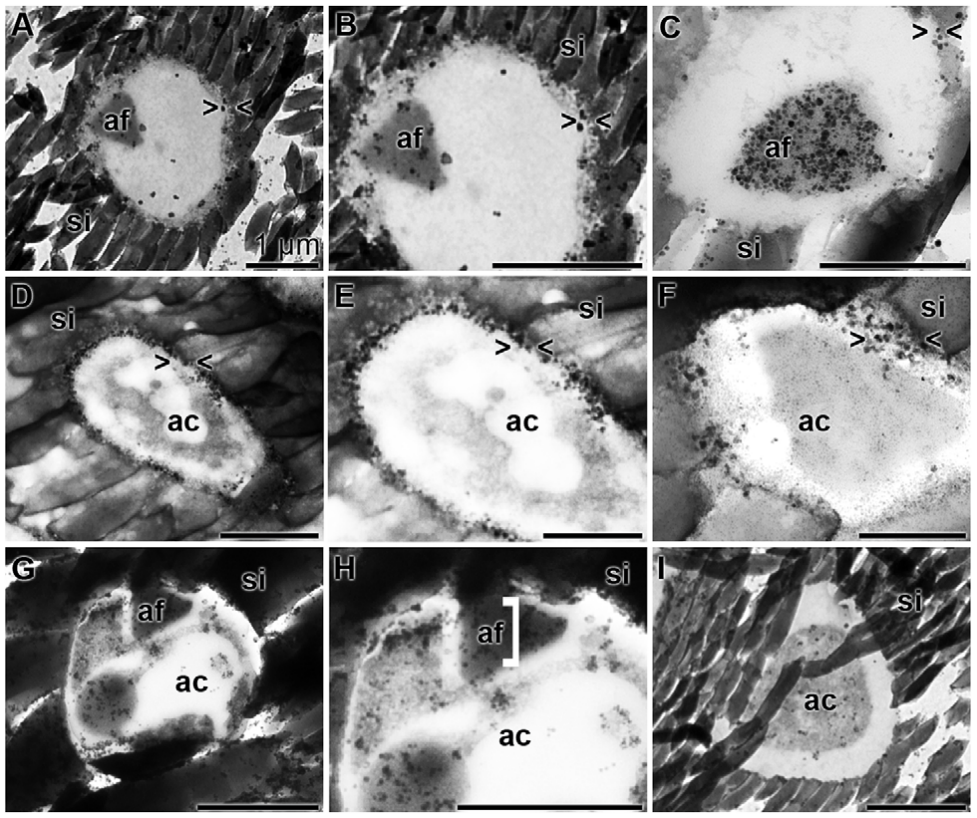
Immunogold labeling electron microscopy [TEM] of the axial canal of spicules. Antibodies against silicatein (**A** to **C**), against aquaporin (**D** to **F**) and against arginine kinase (**G** to **I**) were used. (A to C) The silicatein antibodies reacted with the axial filament (af), which was surrounded by the silica mantel (si), while (D to F) the aquaporin antibodies recognized their antigens primarily at the rim of the axial canal (ac) towards the silica mantel (si). (G to I) The anti-arginine kinase antibodies reacted in a more scattered pattern with the antigen in the axial canal, primarily recognizing membranous structures. The size of all bars represents 1 µm.

TEM analysis has been combined with immunostaining to localize silicatein, aquaporin and arginine kinase within the axial canal (Fig. 7). Arginine kinase was selected as a marker protein for an energy generation (ATP formation); [28]. The fine structure of the axial canal comprised in the more mature phase the axial filament (Fig. 7A). This filament reacted with antibodies raised against silicatein (Fig. 7A to Fig. 7C). It is obvious that not only the filament became decorated with the grains, but also the electron-dense space that surrounded it. In contrast, the reaction of the antibodies against aquaporin was equally strong at the margin of the canal to the silica mantel, the location of the cell membrane (Fig. 7D). At higher magnifications this intense staining was impressive (Fig. 7E and Fig. 7F). In contrast to silicatein, which was mainly found at the axial filament, and of aquaporin, which was found primarily close to the silica mantel, the reaction of the antibodies to the arginine kinase was scattered throughout the axial canal and was predominantly associated with membranous/filamentous structures in the axial canal (Fig. 7G to Fig. 7I).

**Figure 8:**
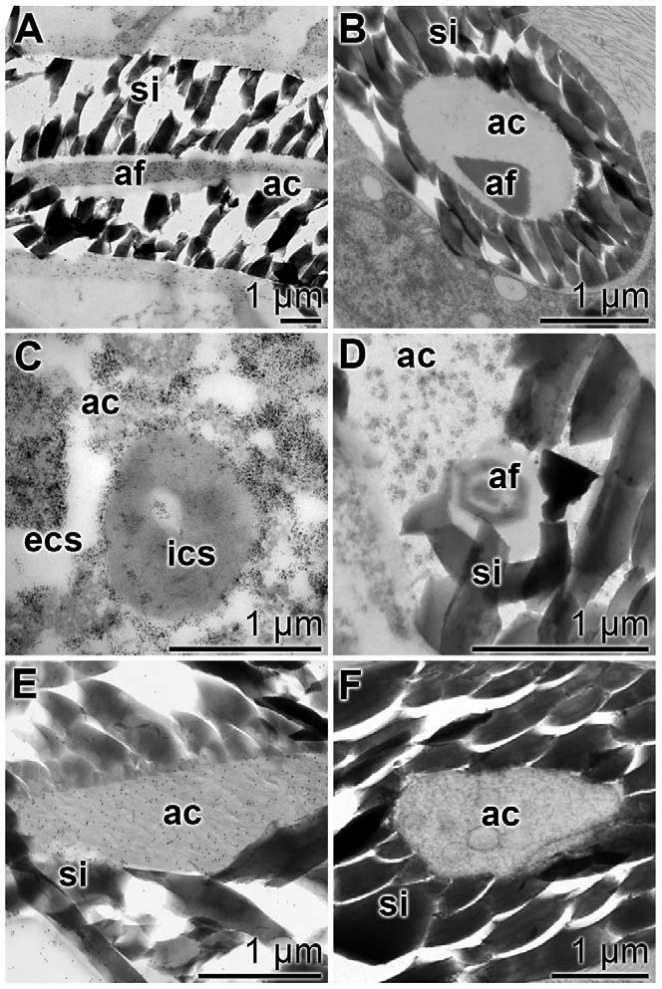
Ultrastructure and immunoelectron microscopy prove the specificity of the antibodies. Sections through spicules were prepared and inspected by TEM analysis. Parallel specimens were reacted either with antibodies or with the preimmune serum kept from this immunization. (**A** and **B**) Silicatein: (A) reaction with PAb-aSILIC_SUBDO; (B) incubation with the corresponding pre-immune serum. (**C** and **D**) Aquaporin: (C) reaction with PoAb-aAQP_SUBDO; (D) corresponding pre-immune serum. (**E** and **F**) Arginine kinase: (E) reaction with PoAb-aAK_SUBDO; (F) corresponding pre-immune serum. The axial canal (ac), the axial filament (af) and the silica shell (si) as well as the intra-cellular space (ics) and the extra-cellular space (ecs) are marked.

In a parallel series of experiments slices were reacted either with the respective antibodies or with the preimmune serum (Fig. 8). A longitudinal section through a spicule was reacted with anti-silicatein and again staining was found primarily to recognize the axial filament (Fig. 8A). In the control, by using preimmune serum, no grains were identified on the axial filament (Fig. 8B). If cross sections through the spicules were incubated with anti-aquaporin the membrane of the cell extension and also the extracellular space to the silica mantel were heavily decorated (Fig. 8C). Again, the preimmune serum did not show any reactivity (Fig. 8D). Likewise, the anti-arginine kinase antibodies reacted over the complete axial canal with the antigens (Fig. 8E), while the pre-immune serum failed to do so (Fig. 8F).

## Discussion

Until this study it remained enigmatic which morphogenetic events trigger and control extracellular axial growth of the siliceous spicules of sponges. Surely the answer to this question will help not only to understand the development of sponge spicules, but may also contribute to the understanding of morphogenesis of more complex skeletal elements, e.g. bone in vertebrates. During bone formation the hydroxyapatite mineral is deposited by osteoblasts apparently in the extracellular space [33]. There, the inorganic deposits are formed to the hydroxyapatite scaffold that is molded into the collagen web [reviewed in: 34]. Morphological studies of the sponge spicules, monaxonal tylostyles, isolated from primmorphs show that their siliceous mantel is composed of a core and a shell cylinder. While the shell is synthesized in the extracellular space by silicatein under consumption of silicate, the core cylinder is formed around the axial canal by the same enzyme/substrate [14]. As presented here, the spicules developing in primmorphs comprise in their axial canals cellular protrusions, with the silicasomes as the most prominent organelles. Those regions of the axial canal are wider [≈2–4 µm] in developing spicules than in canals of spicules from adult specimens [18] or more mature spicules identified in primmorphs [≈0.5 µm]. Especially from longitudinal sections through tylostyles it becomes evident that the wider part of the axial canal of the spicule is directly connected with cells. One cell projection of a sclerocyte reaches into the axial canal of a given growing spicule. The cellular extension ends before the closed tip of the axial canal, the apex, and leaves space for the release of silicasomes and in turn for the discharge of silicatein as well as of silicate. The existence of silicatein in that region has been determined by immuno-TEM, while the presence of silicate was determined by EDX analyses. Both, silicatein and silicate have been identified also in the silicasomes of the sclerocytes localized in the extra-spicular space. Consequently it is compelling to assume that the core cylinder of the spicules is formed by the same enzymatic polycondensation reaction as the one that forms the core cylinder [18]. Silicatein and silicate necessary for both reactions are stored in silicasomes; after translocation of the silicasomes into the extracellular space, they release the two components required for bio-silica synthesis.

Recently we discovered for the first time that the *aquaporin* gene is expressed during spicule formation and contributes to the hardening/aging of the bio-silica product of silicatein (EMBL accession number FR773712 [30]). Sequence similarity analysis revealed that the sponge aquaporin belongs to the group of aquaporins-8. Aquaporin-8 molecules are involved in the absorption of water from the intestine and likewise regulate the intracellular osmo-homeostasis and mucosal fluid fluxes [35]. In turn the sponge aquaporin-8 is the candidate molecule to channel the reaction water into the cells, which is released during the polycondensation of bio-silica in the extracellular space. Sponge cells display a high motility within the bulky extracellular space [25]. Hence, cells that have taken up that reaction water may transport it to more distant places. The consequences are two-fold; (*i*) the removal of water from the site of its formation during the synthesis of bio-silica. In turn, elimination of reaction water causes a shift of the equilibrium towards enhanced silica condensation, resulting in (*ii*) an aging process of the formed bio-silica [36] during which the polymer hardens. By applying antibodies to aquaporin we could demonstrate in the present study that aquaporin exists in two compartments of the axial canal; first, in the silicasome membranes and second, at the cell membranes bordering the silica core of the siliceous mantel. Studies with vertebrate cells revealed that the functional aquaporins are integrated into cell membranes, or into membranes from organelles [37]. In our studies we observed also immune-reactions between aquaporin antibodies and antigens, not associated with membrane structures, which we attribute to reactions with aquaporin pores coming from disintegrating silicasomes.

Based on the TEM studies presented here it is obvious that the silicasomes, both in the extra-spicular and in the intra-spicular space, comprise pores with an approximate size of 1–2 nm. Surely those pores do not reflect aquaporin pores which have a size of around 3 Å [38]. However, the aquaporin channels allowing the transport of small molecules, e.g. water, are usually co-expressed with larger pores (20 to 25 Å) controlling the transport of lower-molecular-weight solutes such as glucose, urea, and creatinine, and also large pores (150 Å) that guide the transport of macromolecules [39]. In view of earlier data [27] and those presented here it can be assumed that water as well as silicate and silicateins are inversely transported through the silicasome membranes and hence the respective channels are preferentially located closely together.

In none of the TEM images clear evidence for the existence of mitochondria could be detected in the cell processes. However, the fast polycondensation reactions [14] are surely energy-consuming [40], [41]. Based on our findings of the absence of mitochondria (to be published) in the 50 µm long cell processes within the axial canal and the results from an earlier contribution that in the presence of silica an upregulation of the ATP generating phosphagen kinase [the arginine kinase] follows, we screened if this kinase is co-localized with the cellular structures within the axial canal. The results indeed revealed an accumulation of the antigens reacting with anti-arginine kinase antibodies within the axial canal structures, assumed to represent the membranes of the silicasomes of the growing spicule.

**Figure 9:**
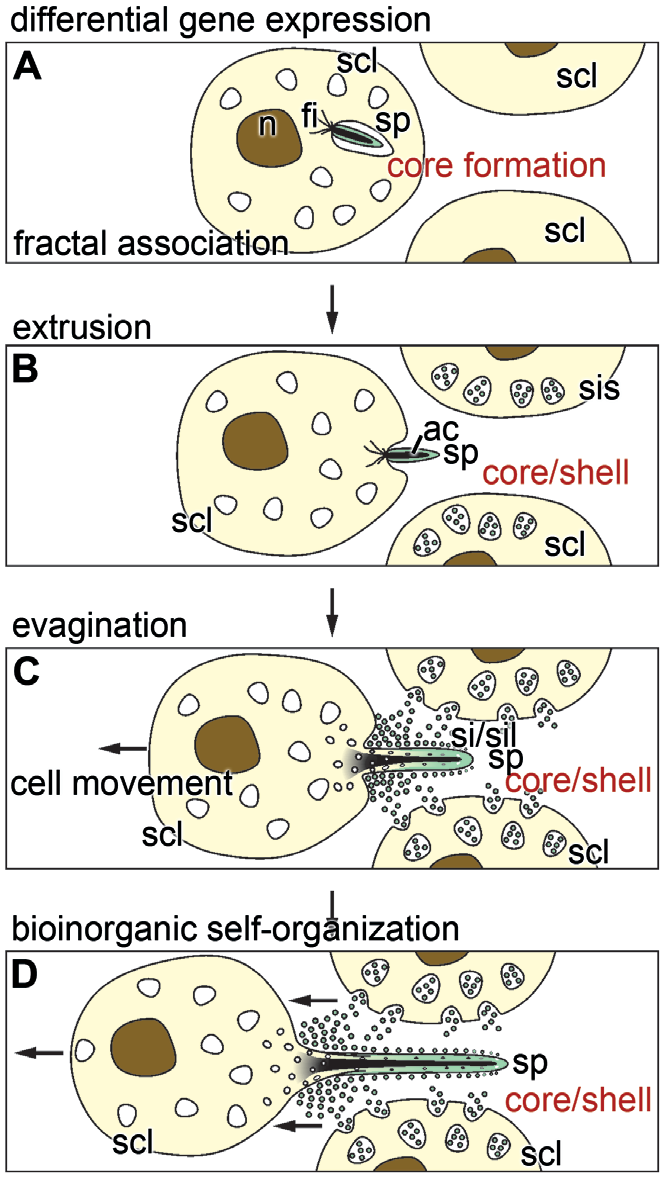
The scheme depicts spicule formation via bio-inorganic self-organization. (**A**) The spicule (sp) synthesis starts intracellularly in sclerocytes (scl). The primordial spicules are associated with filaments (fi) which are assumed to participate in the extrusion of the growing spicule. This phase is dominated by the expression of silicatein that – at the later stage – is required for the formation of both the core and the shell cylinder of the siliceous mantel of the spicule. The newly formed silicatein molecules undergo fractal organization. (**B**) The primordial spicule is extruded and becomes associated in the extracellular space with sclerocytes (scl) which intracellularly form the silicasomes (sis). These organelles contain silicatein and silicate that are released into the extra-spicular space and cause bio-silica formation. (**C**) The growth of the spicule (sp) continues in two directions; axial elongation and appositional growth/thickening. The bio-silica formation is mediated by silicatein (sil) under the consumption of the substrate silicate (si). Growth of spicule is driven both longitudinally and (subsequently) radially along the cell protrusion. During this phase the cell extensions elongate by evagination. The core of the spicule mantel is formed by silicatein, existing in the axial canal, and the shell by silicatein layered onto the outer surface of the growing spicule. (**D**) Final completion of the size and form of the spicule. After termination the spicule disconnects from the sclerocyte (not shown in the scheme) and the hole is closed by bio-silica formation. The direction of cell movement is indicated with an arrow.

Based on the TEM analyses of diagonal and longitudinal sections through growing spicules the most plausible explanation for the dynamics of the extension of the cellular processes into the axial canals of the spicules is to propose hydrodynamic forces that drive an evagination process. Evagination is a frequently occurring process in metazoans, which can be explained by an interplay of movement, intercalation, and division of cells as well as by changes in the cell polarization and shape [42]. On the tissue level evagination is primarily controlled by morphogenetic events that are driven by molecular/genetic processes. A famous example is the Wnt signaling pathway which is controlling development processes but also diseases [43], [44]. This pathway which is based on a sequential expression of a gene cluster is implicated in axis formation in metazoans. We described this pathway in sponges and implied it in the establishment of axis formation in primmorphs and embryos of *S. domuncula* [45]. A chain of differential gene expression causes a controlled cell-cell communication that is essential for the generation of a patterned tissue and/or embryo. It is reasonable to assume that solely cell-based morphogenetic events within tissue or embryos are governed by sequential molecular readout only. However, a quantitatively and also qualitatively different process should be adopted to describe the formation of inorganic/organic structures. Those processes, e.g. here the spicule formation, are driven by a component that is genetically controlled and, in addition, a second component which has to follow hydro-mechanical principles [46], occurring during the purely chemical processes; Fig. 9. The spicule formation is initiated by a sequential gene expression of the *silicateins* and of *collagen*, as described [12]. These gene products are associating to cylinders. Subsequently, a self-organization of silicatein proceeds that is under the control and constraints of fractal parameters [47], [48]. The initial, intracellularly formed primordial spicules are extruded from the cells via evagination, a process which is assumed to be driven by hydro-mechanical forces. These forces are due to differences in the resistance forces of the cell membrane and the forces originating from the intracellular composition of the (macro)molecules and the osmotic pressure. And finally since also inorganic, silica polycondensation reactions are involved, tension forces are occurring during the sol-gel processes of bio-silica formations that guide the cellular extensions and move away the cell body. The latter forces are supposed to occur in a processive way during the evagination of the cell protrusions. This conclusion is also in line with recent observations that during polycondensation reaction both enzymatic and non-enzymatic chemical bonds are formed [49]. As outlined in Fig. 9 the synthesis of the spicule occurs first by axial elongation that results in the bio-silica deposition of the core. Slightly retarded, the process of the appositional growth of the spicules, the shell formation, starts resulting in the thickening of the spicules.

In conclusion, the process of spicule formation can be operationally dissected into the following stages. (*i*) Initial stage of gene expression, primarily of the major spicule-forming gene, *silicatein*, and subsequent association of the formed silicatein molecules following a fractal mechanism (Fig. 9A). (*ii*) Extrusion of the primordial spicule into the extracellular space (Fig. 9B). There, two spatially separated processes of enzymatic polycondensation proceed; first deposition of bio-silica onto the surface of the growing spicules [extra-spicular bio-silica deposition]. The spicule associates with other sclerocytes which in turn transfer their silicasomes into the extracellular space. There, silicatein and its substrate silicic acid are released from those vesicles. Subsequently, bio-silica is formed and deposited onto the surface of the spicule, resulting in an appositional growth. By this, the siliceous shell of the spicule is formed. The second process involves the evagination of the cell protrusion into the axial canal of the spicule where the intra-spicular silicatein-mediated polycondensation process takes place. During this process the core of the silica mantel is formed [intra-spicular bio-silica deposition] (Fig. 9C). This model implies that the cells move away from the growing spicules. It can be suspected that the driving forces required for the cell movement come, at least partially, from the myosin-based motility of the sponge cells [50]. In addition, it is postulated that the process of polycondensation (formation of a rigid silica shell) not only contributes to the elongation of the spicule but also to the separation of the spicule from the sclerocyte body. This process continues until the final form and size of the spicule is completed (Fig. 9D). The remaining hole of the axial canal to the sclerocyte is closed by bio-silica formation, mediated by extra-spicular and also intra-spicular silicatein.

In turn we can summarize the sequence of events that governs spicule formation by the following chain of processes: differential genetic readout (silicatein, collagen) → fractal association of the silicateins → evagination of cells by hydro-mechanical forces → and finally processive polycondensation of bio-silica. We term the interactions of these two processes, biologically driven events and chemically occurring processes that run sequentially or in parallel, as **bio-inorganic self-organization**. This term should express that the bio-genetic self-organization mechanisms, characteristic for the arrangement of cells within tissue, also are under the control of processes that originate from chemical and/or physical processes.
